# Preschoolers’ Understanding of Merit in Two Asian Societies

**DOI:** 10.1371/journal.pone.0114717

**Published:** 2015-05-13

**Authors:** Coralie Chevallier, Jing Xu, Kuniko Adachi, Jean-Baptiste van der Henst, Nicolas Baumard

**Affiliations:** 1 Laboratoire de Neurosciences Cognitives, INSERM U960, DEC, Ecole Normale Supérieure—PSL Research University, Paris, France; 2 Department of Anthropology, Washington University in St. Louis, St. Louis, Missouri, United States of America; 3 Urban-Culture Research Center, Graduate School of Literature and Human Sciences, Osaka City University, Sumiyoshi, Osaka, Japan; 4 Laboratoire Langage, Cerveau et Cognition (L2C2), CNRS, Université de Lyon, UMR 5304, Institut des Sciences Cognitives, Bron, France; 5 Institut Jean-Nicod, UMR 8129, DEC, Ecole Normale Supérieure—PSL Research University, Paris, France; 6 Institute of Cognitive and Evolutionary Anthropology, University of Oxford, Oxford, England, United Kingdom; Lyon Neuroscience Research Center, FRANCE

## Abstract

Recent research in moral psychology have suggested that children make judgments about distributive justice early on in development, and in particular they appear to be able to use merit when distributing the benefits of a collective action. This prediction has recently been validated in various western cultures but it is unknown whether it also applies to more collectivistic cultures, in which the group might be favoured over the individual, and need over merit. Here, we investigate merit-based distributions among 81 children belonging to two Asian societies, China and Japan (mean age = 5.0 years). In line with the idea that children’s moral psychology develops early, we found that Chinese and Japanese children are able to use merit to distribute the benefits of a collective action.

## Introduction

Over the past few years, developmental studies asking children to distribute resources to third parties have consistently demonstrated that the concern for fairness emerges early in development. Infants expect resources to be distributed equally [1,2] and when given the choice between a puppet who performed equal distribution and another puppet who did not, they demonstrate a clear preference for the egalitarian puppet [3]. 3-year-olds share mostly equally after having worked together to obtain rewards in a collaborative task [4]; they take merit into account when distributing the product of common work [5–7] and even in circumstances when they choose not to be fair, they appear to have a good understanding of the fact that they *should* have [8].

Although this work convincingly demonstrates that children’s intuitions about fairness develop early, it is important to note that this work has been carried out in Western populations. It is therefore possible that Western cultural norms have an important impact on the development of fairness and that the early understanding of fairness reported among Western children reflects a local rather than universal phenomenon. In particular, some researchers have argued that Asian societies place more emphasis on collectivism, and emphasize the importance of the group, of attending to others, and of fitting in [9–11]. According to this body of research, these specific norms may promote a different development of fairness principles. Indeed, in this framework, people living in collectivistic societies are claimed to have a different conception of the self and its relation to the group: “In America, ‘the squeaky wheel gets the grease.’ In Japan, ‘the nail that stands out gets pounded down.’ [9]. Since merit is about linking an individual contribution to an individual distribution, these differences in the conceptualization of the self might deemphasize the importance of merit and increase the relevance of equality. Other recent research, however, have emphasized that the distinction between individualism and collectivism has been largely overrated [12,13]. In particular, research on school-age children living in Hong Kong [14], Brazil [15] and in many countries commonly described as collectivistic has provided evidence of both cross-cultural similarities and within-culture variation in fairness judgments. More generally, school-age children in a range of cultures conceived as collectivistic have been found to value individual rights [16], Wainryb’s work on moral judgments and reasoning with the Druze in Israel [17], Killen and collaborators’s research on fairness judgments in Colombia and Japan [18,19], Turiel’s research on fairness judgments in Turkey [20].

In this paper, we examined preschoolers’ judgments regarding the distribution of resources according to merit (i.e. individual contribution) in two Asian societies (Japan and China). We chose these two Asian societies because they have different religious backgrounds (mainly Shintoism and Buddhism in Japan, mainly atheism in China) and different wealth levels (Japan’s GDP per capita being among the highest in the world and China’s being slightly below the world’s average). Our goal was to assess whether children living in these two societies have a preference for distributions based on merit vs. on equality and whether, regardless of their preferences, they have the ability to take merit into account in a forced-choice situation. With this goal in mind, we adapted Baumard et al.’s design [5], in which children are asked to distribute three cookies to two characters who did not work equally hard to bake the cookies. The experiment involves two stages: an initial distribution phase and a final distribution. During the initial distribution phase, children may distribute any number of cookies they want, in any way they want. In the final distribution, however, children have to distribute any remaining cookie, which thus forces them to favor one character over the other. This two-step design therefore allows us to study cross-cultural differences both in children’s moral *preferences* and in their *capacity* to take merit into account. For example, children might *prefer* to give each character one cookie in the initial phase but if they have the underlying *capacity* to take merit into account, they should favor the big contributor once forced to distribute the remaining cookie(s) in the final phase. Using this design, Baumard et al. [5] found that Western preschoolers (3 to 5 year-olds) have an initial preference for equal distributions but their final distribution reflects that they nonetheless understand that the greater contributor has a right to a greater share than the smaller contributor.

In line with the idea that the distinction between collectivism and individualism does not predict children’s psychological development, we anticipated that children brought up in collectivistic societies would behave similarly to Western children. That is, we predicted that children’s modal response in the free distribution phase would be egalitarian but that the forced choice phase would reveal their underlying understanding of merit, with the big contributor favored over the small contributor.

## Methods

### Participants

81 children were included in this study: 39 in Japan (mean: 61 months, range 51–67 months, 22 girls) and 42 in China (mean: 59 months, range 51–67 months, 22 girls). An additional 4 Japanese children (aged 69–70 months) were excluded from the sample in order to keep the age ranges identical in both cultures.

### Japanese Setting

The experiment was carried out in a kindergarten in Osaka. The experimenter was familiar with the school, which she had visited before. The experimenter participated in the daily morning activities in the preschool so that she familiarized herself with all the participants. Participants were tested individually in a quiet room they were familiar with, close to their own classroom. The experiment was conducted in Japanese, the mother tongue of both the participants and me.

### Chinese Setting

The experiment was conducted as part of the experimenter’s ethnographic fieldwork at a middle-class private preschool in March 2012, Shanghai, China. By the time the experiment took place, the experimenter had already done field-research in that preschool on a daily basis for more than half a year and familiarized herself with all the participants. Participants were tested individually in a quiet room they were familiar with, close to their own classroom. The experiment was conducted in Mandarin Chinese, the mother tongue of both the experimenter and the participants.

### Ethics Statement

The procedure was approved by the local Institutional Review Board at Washington University (for Chinese participants) and Osaka City University (for Japanese participants) and complied with local human research policies. Children were recruited from local schools and were only included in the study if parents had provided written consent before the experimenter’s visit. Children gave informed assent at the beginning of the procedure. Participants were tested individually in a quiet room close to their classroom.

### Procedure

**Fig 1:**
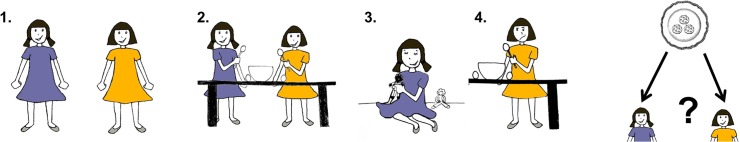
Example of a set of vignettes used in the experiment (other vignette sets were available so that the identity of the big contributor could be counterbalanced across participants. Pictures 1, 2 and 5 remained constant).

The experimenter introduced the characters of the story, saying: *“See*, *this is Yuki/Lele (Japan/China)*, *and this is Mika/JingJing (Japan/China)*. *Yuki/Lele and Mika/JingJing are very good friends*. *Can you show me Mika/JingJing*? *Can you show me Yuki/Lele*?*”* The experimenter ensured that the child had memorized the names by asking the child to point to Yuki/Lele (right) and to Mika/JingJing (left; see Fig 1, Picture 1). The experimenter turned to Picture 2 and said *“Today*, *Yuki/Lele and Mika/JingJing have decided to bake cookies*. *See*, *they’re in the kitchen; they’re making cookies*! *After a little while*, *Mika/JingJing is bored with making the cookies*. *Yuki/Lele says*, *“Yes*, *it’s tiring to bake cookies*, *but I’m OK to finish on my own*.*”* The experimenter then showed the third picture and said: *“Mika/JingJing goes to play with her doll*. *Yeepee*! *It’s fun to play dolls*!*”* At this point, the first control question was asked: *“Does Mika/JingJing find it fun to play dolls*?*”* If the child provided the correct answer, the experimenter then proceeded to the fourth picture, saying, *“In the meantime*, *Yuki/Lele is finishing the cookies*. *She says*, *‘Phew*!! *This is such hard work*! *It’s so tiring to make these cookies*!*’ Yuki/Lele is working really hard*.*” The second control question was asked*: *“Does Yuki/Lele find that it’s a lot of work to bake the cookies*?*”* If the correct answer was provided, the experimenter showed the final picture and said *“That’s it*! *Yuki/Lele is done*! *The cookies are ready*! *Mum says*, *‘You can have some*!*’ [Experimenter puts three same-sized cookies on a plate*.*] You can give cookies to Yuki/Lele and Mika/JingJing*.*”*

A picture displaying Yuki/Lele and Mika/JingJing’s faces, was shown again so that the children could distribute the cookies. The experimenter then waited 10 seconds or for a clear sign from the child that she had finished distributing (e.g., “I’m done”). The “initial” distribution was recorded at this point. If the child had not distributed all the cookies, the experimenter went on saying, *“Well done*! *Very nice*! *Oh*, *look*, *there’s some left*. *Who do you want to give it to*? *To Yuki/Lele or to Mika/JingJing*?*”* (order of names counterbalanced) and repeated the procedure until all cookies were given out (children could thus distribute the cookies in one, two, or three steps). The “final” distribution was recorded at this point. Importantly, children were not given the option of maintaining an equal distribution at this stage (their distribution thus does not reflect their spontaneous preferences but rather their ability to consider that merit is a relevant factor in a forced choice situation). Finally, after the child had made her choice, she was explicitly asked to justify her answer. To do so, the experimenter asked “*Why*?*”* after the child indicated whether it was better to give the big cookie to *Yuki/Lele* or to *Mika/JingJing*.

## Results

**Fig 2:**
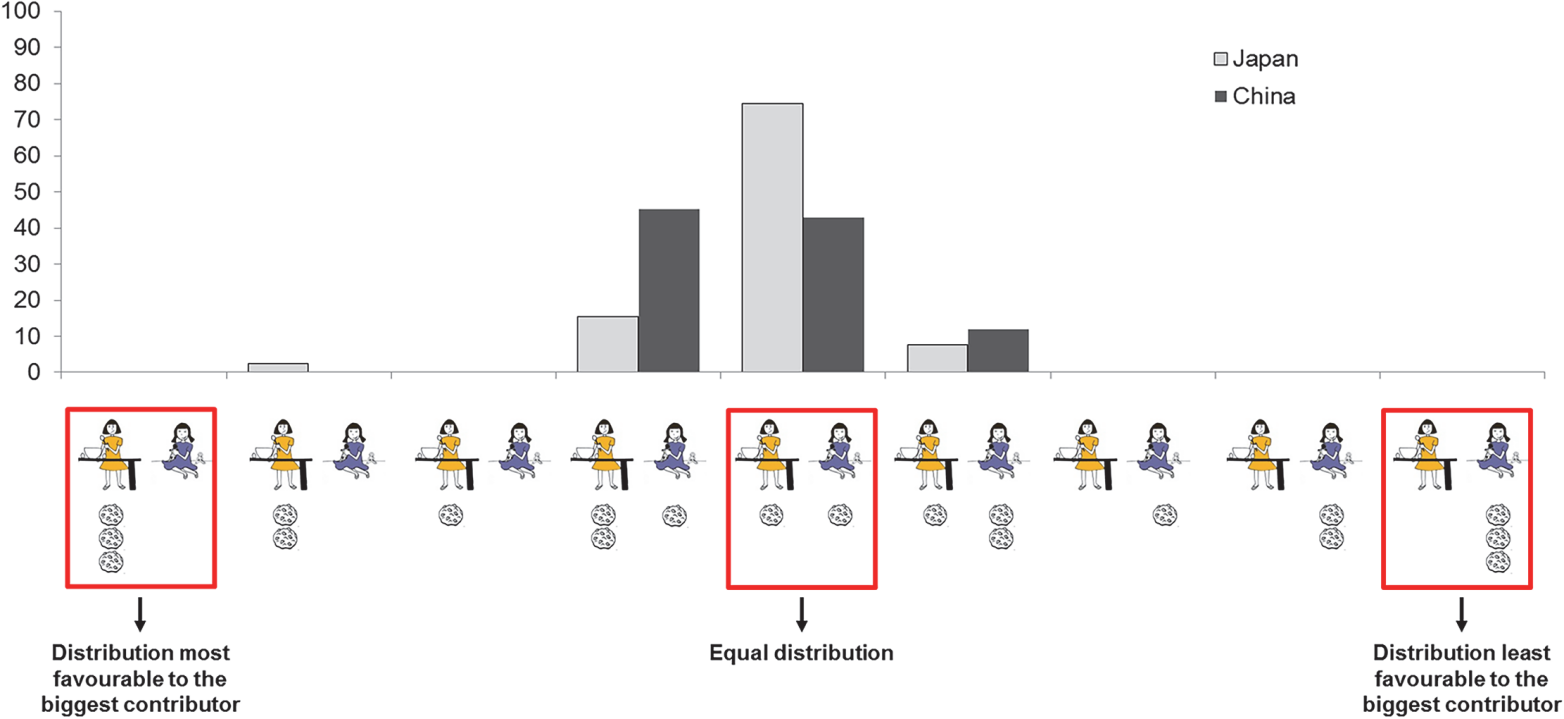
Pattern of initial distribution among Japanese children (grey) and Chinese children (black).

**Fig 3:**
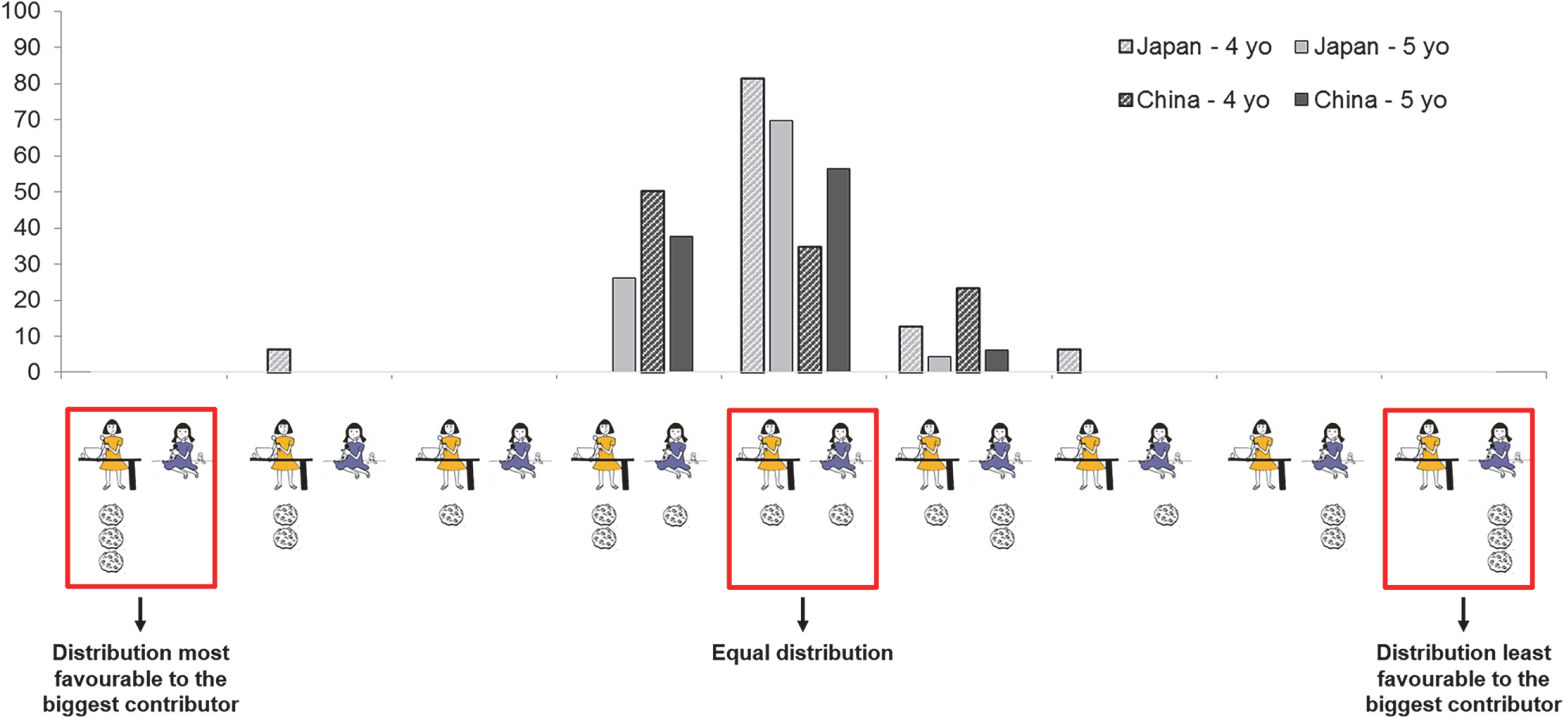
Pattern of initial distribution among Japanese children (grey) and Chinese children (black) split by age groups (4 year olds hatched, 5 year olds plain).

The full anonymized dataset is available in the S1 File. In the free distribution phase, Japanese children’s modal response was egalitarian, with 29 out of 39 children distributing one cookie to each character and the second most frequent distribution, chosen by 6 children, was one that favoured the character who had contributed more to the cooking (i.e., two cookies for the big contributor and one for the small contributor) (see Fig 2). Among the 42 Chinese children, by contrast, a clear bimodal response pattern was observed, with 18 children choosing an egalitarian distribution and 19 children favoring the big contributor. A Fisher’s exact test comparing the two most frequent distributions amongst Japanese vs. Chinese children revealed that this cross-cultural difference in the initial distribution phase was indeed significant, Fisher’s exact test *p* = .03, OR = 3.06. We then divided the sample in two age groups: a younger group (4 year-olds) and an older group (5 year-olds) and found that this difference between Japan and China was stronger in the younger age group with no Japanese vs. half the Chinese Japanese 4-year olds giving two cookies to the big contributor and one to the small contributor (see Fig 3).

**Fig 4:**
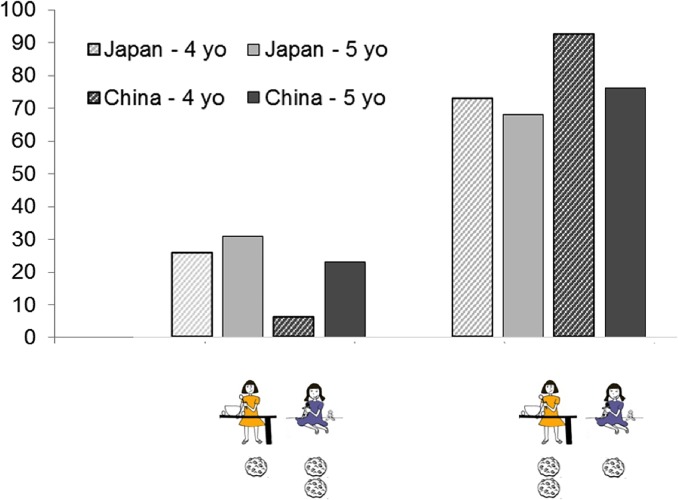
Pattern of final distributions split by country (Japan in grey, China in Black) and age group (4 year olds hatched; 5 year olds plain).

In the forced distribution phase however, children in both societies favored the big contributor (see Fig 4): 28 out of 39 children in Japan (i.e., 72%), two-choice binomial test *p* = .01, OR = 2.54; 35 out of 42 children in China (i.e., 83%), two-choice binomial test *p* < .001, OR = 5. The forced distribution did not differ between Chinese and Japanese children, Fisher’s exact test *p* = .74. The two age groups did not differ in their final distribution, Fisher’s exact test *p* = .28. Moreover, we confirmed that the pattern identified in the whole sample remained when the younger sample was considered separately, with a total of 31 out of 43 children favoring the big contributor, two-choice binomial test *p* = .005, OR = 2.59. Finally, children were asked to justify why they picked their specific final distribution. Following the procedure used in Baumard et al. (2012), justifications mentioning the characters’ respective levels of contribution were considered correct (e.g., “Because Yuki prepared more of the cake”). Other justifications (e.g., “Because I like it”) or an absence of justification (“Don’t know” or silence) was coded as incorrect. A second coder classified the children’s justifications. Agreement between coders was 100%. Strikingly, 48 children provided correct justifications for their choices and only 12 provided incorrect justifications; 21 children did not respond (see Table 1 and S1 File for details).

**Table 1:** Number of correct, incorrect and absent justifications split by age group and culture.

	Correct	Incorrect	No response
Japan—4 yo	6	6	4
Japan—5 yo	13	4	6
China—4 yo	18	1	7
China—5 yo	11	1	4
**TOTAL**	**48**	**12**	**21**

## Discussion

In this study, we demonstrated that children brought up in two Asian societies, China and Japan, are able to take merit into account when distributing a commonly produced good. Indeed, children consistently favoured the biggest contributor in their final distribution, which demonstrates their ability to match contribution and distribution. When compared to existing data collected using the same procedure [5], Asian children’s understanding of merit therefore appears quite similar to that of Western children, who were 74% to favour the big contributor in the final distribution (in comparison, 72% of Japanese children and 83% of Chinese children favored the big contributor in our experiment). This suggests that, in spite of cross-cultural variations in levels of collectivism, children’s fairness behaviour are guided by common underlying principles.

The initial distribution phase, however, revealed cross-cultural differences in children’s spontaneous preferences: Japanese children (much like Western children tested using the same procedure [5]) consistently favored an egalitarian distribution but this initial egalitarian preference was less frequent among Chinese children. We had no prior expectations that Japanese and Chinese children would differ in this initial distribution phase but we can speculate on a range of factors which may explain this phenomenon. First, recent ethnographic work notes that merit is an important construct in contemporary China [21]. In particular, Chinese schools—in contrast to Japanese schools—emphasize performance based rewards among children, as well as teachers. Informal interviews led by the second author in the school where the research was conducted indeed revealed that very young children are already infused with the idea that whoever does well at school deserves a better reward[21] [21]. To Chinese preschoolers, it might therefore be quite natural to consider that different performance call for different rewards.

Second, China and Japan are in different socioeconomical situations. Although our study did not test this factor directly, it is possible that socioeconomic status (SES) has an impact on children’s distributive preferences. Recent work on adults living in different socioeconomic environments within the same culture has indeed demonstrated that SES has an impact on moral psychology [22]. In addition, Japan and China also differ on overall trust levels—Japan being a high trust country and China a low trust country [23], which might have a broad influence on cooperative behaviours [24]. Specifically, cooperative interactions are sometimes not immediately beneficial and the costs engaged in a given interaction are sometimes only offset over the longer run. Distributions which place a given individual at a slight disadvantage are therefore more likely to be tolerated when individuals have evidence that they are involved in a long term interaction. In high trust environment, for instance, people can choose to share the benefits of cooperation equally even when this puts them at a slight disadvantage because they can assume that individual costs will even out in the long term. If the distribution is skewed in favor of one cooperator in the short term, it will remain mutually beneficial in the long term [25]. When individuals are less sure of their long term perspectives, however, a better solution might be to immediately share the benefits of cooperation in proportion to each individual’s contribution. This solution allows to avoid situations in which the interaction stops before a productive contributor has been properly compensated for its contribution. Future cross-cultural work should therefore address these apart these various factors in order to gain a finer understanding of within and between cultures differences in children’s moral judgments.

A number of other limitations should also be acknowledged at this point. First, although relatively standard in cross-cultural research, the size of each sample once children are split by age and culture is relatively small. Second, while the present study clearly demonstrates that children are able to consider merit in the forced-choice phase, it is unclear whether they see their choice as the best option in terms of fairness or as a way to make the best of a bad situation (i.e., they might consider that giving two cookies to the big contributor is better than giving two cookies to the small contributors without going so far as to thinking that this forced distribution is fair). Moreover, although this interpretation is unlikely based on the justifications we collected, it is possible that at least some children chose the deserving character for reasons unrelated to fairness (e.g., because they happen to like hard workers better, and want to allocate more resources to individuals they like).

## Supporting Information

S1 File (PDF)
