# Supplementary material for: Preschoolers’ Understanding of Merit in Two Asian Societies
Source: PLoS One. 2015 May 13;10(5):e0114717. doi: 10.1371/journal.pone.0114717 (PMC4430231; doi:10.1371/journal.pone.0114717)
Supplement: S1 File — (PDF) [file pone.0114717.s001.pdf]

Country	Name	Age (months)	School level	Sex	who works?	Cookie 1 (Girl 1/Girl2)	Cookie 2 (Girl 1/Girl2)	Cookie 3 (Girl 1/Girl2)	Cookie 2or3 (Girl 1/Girl2)	Initial distribution B=Big contrib. S=Small contrib	Final distribution B=Big contrib. S=Small contrib	Final Distributio n 0 vs 1	Justifications	Final Justif 0 wrong, 1 correct, 2 no answer DK,
Japan	Jpy20	51	junior	f	M	Y	M		M	1B/1S	2B/1S	1	One cookie was left. I gave it to Mika.	0
Japan	Jpy12	54	middle	m	M	M	Y		M	1B/1S	2B/1S	1	Mika did her best.	1
Japan	Jpy7	54	junior	m	M	Y	M		M	1B/1S	2B/1S	1	Mika did her best alone.	1
Japan	Jpy6	54	junior	m	M	M	Y		M	1B/1S	2B/1S	1	I thought that Mika has had better take two.	0
Japan	Jpy21	55	junior	f	M	M	Y		Y	1B/1S	1B/2S	0	Yuki stopped making cookies on the way.	1
Japan	Jpy17	55	junior	f	M	Y	Y	M		1B/2S	1B/2S	0	Mika made cookies so hard. Yuki did't make them.	1
Japan	Jpy5	55	junior	m	M	M	Y		M	1B/1S	2B/1S	1	I don't know	2
Japan	Jpy4	55	junior	m	M	M	Y		M	1B/1S	2B/1S	1	I don't know	2
Japan	Jpy3	56	junior	m	M	Y	M		Y	1B/1S	1B/2S	0	Yuki likes cookies.	0
Japan	Jpy19	57	junior	f	M	Y	M		M	1B/1S	2B/1S	1	Mika worked very hard.	1
Japan	Jpy18	58	junior	f	M	Y	M		M	1B/1S	2B/1S	1	This was the best way.	0
Japan	Jpy2	59	junior	m	M	M	Y		Y	1B/1S	1B/2S	0	This looked good.	0
Japan	Jpy1	59	junior	m	M	Y	M		M	1B/1S	2B/1S	1	I don't know	2
Japan	Jpy30	59	middle	f	M	Y	Y	M		1B/2S	1B/2S	0	This was the best way.	0
Japan	Jpy29	59	middle	f	M	Y	M		M	1B/1S	2B/1S	1	Mika made cookies until the end	1
Japan	Jpy16	60	middle	m	M	M	M		Y	2B/0S	2B/1S	1	(He was not available to comment.)	2
Japan	Jpy28	60	middle	f	M	M	Y		Y	1B/1S	1B/2S	0	This was the best way.	0
Japan	Jpy11	61	middle	m	M	M	Y		Y	1B/1S	1B/2S	0	Yuki did't make cookies.	1
Japan	Jpy10	62	middle	m	M	M	Y	M		2B/1S	2B/1S	1	Mika made cookies until the end	1
Japan	Jpy25	62	middle	f	M	Y	M	M		2B/1S	2B/1S	1	Mika made cookies until the end	1
Japan	Jpy27	62	middle	f	M	Y	M		Y	1B/1S	1B/2S	0	One piece of cookie was short to distribute them fairly.	0
Japan	Jpy26	62	middle	f	M	Y	M		M	1B/1S	2B/1S	1	The third cookie was left. Yuki played with the doll, Mika made cookies.	1
Japan	Jpy15	63	middle	m	M	Y	M	Y		1B/2S	1B/2S	0	This was the best way.	0
Japan	Jpy14	63	middle	m	M	Y	M		M	1B/1S	2B/1S	1	Mika did her best.	1
Japan	Jpy36	64	middle	f	M	M	Y		M	1B/1S	2B/1S	1	Mika made cookies. So I gave her two.	1
Japan	Jpy34	64	middle	f	M	M	Y		M	1B/1S	2B/1S	1	I don't know	2
Japan	Jpy23	64	middle	f	M	Y	M	M		2B/1S	2B/1S	1	Yuki did her best a little. On the other hand, Mika did her best.	1
Japan	Jpy13	64	middle	m	M	M	Y	M		2B/1S	2B/1S	1	(He was not available to comment.)	2
Japan	Jpy35	65	middle	f	M	Y	M		M	1B/1S	2B/1S	1	This looked good.	0
Japan	Jpy22	65	middle	f	M	M	Y		M	1B/1S	2B/1S	1	The third cookie was left, and there was no help for it. Mika made cookies very hard	1
Japan	Jpy9	65	middle	m	M	M	Y		Y	1B/1S	1B/2S	0	(He was not available to comment.)	2
Japan	Jpy33	65	middle	f	M	Y	M		M	1B/1S	2B/1S	1	Mika did her best. So I gave her two.	1
Japan	Jpy32	65	middle	f	M	M	Y		M	1B/1S	2B/1S	1	Yuki did't make cookies. Mika made cookies until the end	1
Japan	Jpy31	65	middle	f	M	M	Y		M	1B/1S	2B/1S	1	I'd like to give the last cookies to Mika.	2
Japan	Jpy8	66	middle	m	M	M	Y		Y	1B/1S	1B/2S	0	I don't know	2
Japan	Jpy24	66	middle	f	M	M	Y	M		2B/1S	2B/1S	1	This was the best way.	2
Japan	Jp39	66	middle	f	M	Y	M		M	1B/1S	2B/1S	1	Because Mika did its best until the end	1
Japan	Jp34	66	middle	f	M	Y	M	M		2B/1S	2B/1S	1	Because Mika did its best, she gets two.	1
Japan	Jp12	67	middle	m	M	Y	M		M	1B/1S	2B/1S	1	Because Mika tried the making of cake hard until the end,it should be given her	1
China	Ch1	51	3	M	Girl 1	Girl 1	Girl 2	Girl 1		2B/1S	2B/1S	1	Because she finished making the cookies.	1
China	Ch2	51	2	F	Girl 1	Girl 1	Girl 2	Girl 1		2B/1S	2B/1S	1	Girl 1 finished making cookies, so I gave her two.	1
China	Ch3	51	1	M	Girl 2	Girl 1	Girl 2		Girl 1	1B/1S	1B/2S	0	I want to give the last cookie to Girl 2. Because she made cookies on her own.	1
China	Ch4	52	3	F	Girl 2	Girl 2	Girl 1		Girl 2	1B/1S	2B/1S	1	Because she finished making the cookies, and it was very tiring.	1
China	Ch5	53	3	M	Girl 2	Girl 1	Girl 2	Girl 2		2B/1S	2B/1S	1	Because Girl 2 made cookies.	1
China	Ch6	53	2	M	Girl 2	Girl 2	Girl 1	Girl 1		1B/2S	1B/2S	0	(no response)	2
China	Ch7	54	3	F	Girl 1	Girl 1	Girl 2	Girl 1		2B/1S	2B/1S	1	Girl 1 worked more.	1
China	Ch8	54	3	M	Girl 1	Girl 1	Girl 2		Girl 1	1B/1S	2B/1S	1	(remained silent)	2
China	Ch9	54	3	F	Girl 1	Girl 1	Girl 2	Girl 1		2B/1S	2B/1S	1	Because Girl 1 loves cookies!	0
China	Ch10	54	3	M	Girl 2	Girl 1	Girl 2	Girl 1		1B/2S	1B/2S	0	I don't know	2
China	Ch11	54	3	M	Girl 1	Girl 2	Girl 1	Girl 1		2B/1S	2B/1S	1	Because she worked more. Actually she worked a lot!	1
China	Ch12	54	3	M	Girl 1	Girl 1	Girl 2		Girl 1	1B/1S	2B/1S	1	Girl 2 went to play dolls, and Girl 1 finished making all the cookies.	1
China	Ch13	54	2	M	Girl 2	Girl 1	Girl 2		Girl 2	1B/1S	2B/1S	1	Because Girl 2 continued making cookies, while Girl 1 went to play dolls.	1
China	Ch14	54	2	M	Girl 2	Girl 1	Girl 2		Girl 1	1B/1S	1B/2S	0	I don't know	2
China	Ch15	54	2	F	Girl 2	Girl 1	Girl 2	Girl 1		1B/2S	1B/2S	0	(no response)	2
China	Ch16	54	2	F	Girl 1	Girl 1	Girl 2		Girl 1	1B/1S	2B/1S	1	Because Girl 1 made cookies!	1
China	Ch17	56	3	F	Girl 2	Girl 2	Girl 1	Girl 2		2B/1S	2B/1S	1	Because Girl 2 insisted on making all the cookies.	1
China	Ch18	57	3	F	Girl 2	Girl 1	Girl 2	Girl 2		2B/1S	2B/1S	1	Because Girl 2 worked more.	1
China	Ch19	57	3	M	Girl 2	Girl 2	Girl 1	Girl 1		1B/2S	1B/2S	0	(he didn't answer)	2
China	Ch20	57	3	M	Girl 1	Girl 1	Girl 2	Girl 1		2B/1S	2B/1S	1	Because she finished making the cookies, and it was really tiring.	1
China	Ch21	57	3	F	Girl 1	Girl 1	Girl 2	Girl 1		2B/1S	2B/1S	1	Because Girl 1 finished making the cookies while Girl 2 played dolls and didn't finish	1
China	Ch22	58	3	F	Girl 2	Girl 2	Girl 1		Girl 2	1B/1S	2B/1S	1	Because Girl 2 worked more.	1
China	Ch23	58	3	F	Girl 2	Girl 1	Girl 2	Girl 2		2B/1S	2B/1S	1	Because Girl 1 went to play dolls and Girl 2 continued to make all the cookies.	1
China	Ch24	59	3	F	Girl 1	Girl 1	Girl 2	Girl 1		2B/1S	2B/1S	1	(no response)	2
China	Ch25	59	3	F	Girl 2	Girl 1	Girl 2		Girl 2	1B/1S	2B/1S	1	Because Girl 1 is a free-rider.	1
China	Ch26	59	3	M	Girl 1	Girl 1	Girl 1	Girl 1		2B/1S	2B/1S	1	All three to Girl 1, because Girl 2 went out to play.	1
China	Ch27	61	3	F	Girl 2	Girl 1	Girl 2	Girl 2		2B/1S	2B/1S	1	Because Girl 2 worked more and Girl 1 worked less.	1
China	Ch28	61	3	M	Girl 1	Girl 1	Girl 1	Girl 2		2B/1S	2B/1S	1	Because Girl 2 went to play toys.	1
China	Ch29	62	3	M	Girl 2	Girl 1	Girl 2	Girl 1		1B/2S	1B/2S	0	(no response)	2
China	Ch30	63	3	M	Girl 2	Girl 2	Girl 1		Girl 2	1B/1S	2B/1S	1	I don't know	2
China	Ch31	63	3	F	Girl 1	Girl 1	Girl 2		Girl 1	1B/1S	2B/1S	1	Because it's very tiring for Girl 1 to make all the cookies.	1
China	Ch32	63	3	M	Girl 2	Girl 2	Girl 1		Girl 2	1B/1S	2B/1S	1	Because she finished making the cookies!	1
China	Ch33	64	4	F	Girl 2	Girl 1	Girl 2		Girl 2	1B/1S	2B/1S	1	Because Girl 1 left in the middle of making cookies.	1
China	Ch34	65	4	M	Girl 1	Girl 2	Girl 1	Girl 1		2B/1S	2B/1S	1	I don't know	2
China	Ch35	65	4	M	Girl 2	Girl 2	Girl 1		Girl 2	1B/1S	2B/1S	1	Because Girl 2 sticked to making cookies and finished the job. It is good to be persi	1
China	Ch36	65	4	F	Girl 1	Girl 1	Girl 2		Girl 1	1B/1S	2B/1S	1	I asked her why she gave two to Girl 1 and one to Girl 2, she remained silent for a	2
China	Ch37	66	3	F	Girl 1	Girl 1	Girl 2	Girl 1		2B/1S	2B/1S	1	Because Girl 1 worked more.	1
China	Ch38	66	3	M	Girl 1	Girl 1	Girl 1	Girl 2		2B/1S	2B/1S	1	Because Girl 2 didn't make cookies together with Girl 1.	1
China	Ch39	67	4	F	Girl 1	Girl 1	Girl 2		Girl 1	1B/1S	2B/1S	1	Because she did a better job.	1
China	Ch40	67	4	F	Girl 2	Girl 1	Girl 2		Girl 2	1B/1S	2B/1S	1	Because she made cookies!	1
China	Ch41	67	4	F	Girl 2	Girl 1	Girl 2		Girl 2	1B/1S	2B/1S	1	They wanted to make cookies, but Girl 1 went to play dolls, and Girl 2 finished mak	1
China	Ch42	67	4	F	Girl 1	Girl 2	Girl 1	Girl 1		2B/1S	2B/1S	1	Because Girl 2 is older.	0
